# Mutational disruption of the *ABCC2* gene in fall armyworm, *Spodoptera frugiperda*, confers resistance to the Cry1Fa and Cry1A.105 insecticidal proteins

**DOI:** 10.1038/s41598-018-25491-9

**Published:** 2018-05-08

**Authors:** Lex Flagel, Young Wha Lee, Humphrey Wanjugi, Shilpa Swarup, Alana Brown, Jinling Wang, Edward Kraft, John Greenplate, Jeni Simmons, Nancy Adams, Yanfei Wang, Samuel Martinelli, Jeffrey A. Haas, Anilkumar Gowda, Graham Head

**Affiliations:** 1 Monsanto Company, Chesterfield, Missouri USA; 2 Monsanto Company, Cambridge, Massachusetts, USA; 3 Monsanto Company, Union City, Tennessee USA; 4 Monsanto Company, Creve Coeur, Missouri USA; 5 Genentech, South San Francisco, California, USA

## Abstract

The use of Bt proteins in crops has revolutionized insect pest management by offering effective season-long control. However, field-evolved resistance to Bt proteins threatens their utility and durability. A recent example is field-evolved resistance to Cry1Fa and Cry1A.105 in fall armyworm (*Spodoptera frugiperda*). This resistance has been detected in Puerto Rico, mainland USA, and Brazil. A *S. frugiperda* population with suspected resistance to Cry1Fa was sampled from a maize field in Puerto Rico and used to develop a resistant lab colony. The colony demonstrated resistance to Cry1Fa and partial cross-resistance to Cry1A.105 in diet bioassays. Using genetic crosses and proteomics, we show that this resistance is due to loss-of-function mutations in the *ABCC2* gene. We characterize two novel mutant alleles from Puerto Rico. We also find that these alleles are absent in a broad screen of partially resistant Brazilian populations. These findings confirm that *ABCC2* is a receptor for Cry1Fa and Cry1A.105 in *S. frugiperda*, and lay the groundwork for genetically enabled resistance management in this species, with the caution that there may be several distinct *ABCC2* resistances alleles in nature.

## Introduction

The fall armyworm, *Spodoptera frugiperda* (J.E. Smith) (Lepidoptera: Noctuidae), is one of the most significant crop pests across a vast area in North and South America. This polyphagous species feeds on major crops such as maize, soybean, cotton, and sugarcane, in addition to a wide variety of other plant species^1^. Severe infestations of *S. frugiperda* can be devastating, resulting in significant crop loss^2^. This species is also highly mobile, for example, in North America, populations overwinter in the southern United States and migrate as far north as Canada in the summer months. Moreover, *S. frugiperda* is highly adaptive and has developed resistance to numerous classes of chemical insecticides^3^, and more recently to plant-expressed Bt proteins^4–6^.

The use of plant-expressed Bt proteins (also known as Bt crops) is a significant advance in *S. frugiperda* control. These proteins are stably transformed into a crop genome and when expressed make the plant tissues toxic to target insects after feeding. In particular, certain Bt proteins belonging to the Cry1 and Cry2 families have proven to be highly effective in controlling *S. frugiperda*^7^, including in particular Cry1Fa, Cry1A.105 and Cry2Ab2, and to a lesser extent Cry1Ab. The use of these proteins for *S. frugiperda* control has become widespread in maize and cotton. With high utilization in Bt crops, the Bt proteins come under increased selection pressure and increased risk of resistance in target insects, including *S. frugiperda*. For example, reports of *S. frugiperda* resistance to Cry1Fa first surfaced in maize grown in Puerto Rico in 2006, and were later confirmed in 2010^5,6^. This resistance issue was severe enough that it led to the removal of products containing the Cry1Fa expressing TC1507 transgenic event from the Puerto Rican market^5^. Additionally, Cry1Fa resistant insects were also found to be partially cross-resistant to other Cry1 family proteins, including Cry1Ab and Cry1Ac^5^, and Cry1Aa^8^, suggesting a shared mode of action for these Cry1 family proteins. This result has been verified biochemically, with the addition of Cry1A.105 as another protein with a high potential for cross-resistance with Cry1Fa^9^. More recently, selection experiments using purified Cry1A.105 also indicated that increased resistance to that protein lead to a coordinated increase in resistance to Cry1Fa^10^, again highlighting a potential for cross-resistance between Cry1Fa and Cry1A.105.

Following this initial discovery of resistance in Puerto Rico, additional studies have reported resistance to Cry1Fa in Brazil^11^ and the Southeastern US^4^. In both studies the resistance allele was found to be recessive and capable of delivering at least 84-fold resistance relative to a susceptible control population. The study by Huang *et al*.^4^ also demonstrated empirically that there is partial cross-resistance between Cry1Fa and Cry1A.105, as earlier biochemical assays had predicted^9^.

The development of resistance to members of the Cry1 family of toxins threatens their continued use in controlling *S. frugiperda*. Insect Resistance Management (IRM) programs have been implemented to fight the spread of resistance. IRM seeks to develop pest management practices that maintain low resistance allele frequency and minimize the probability of resistance spreading to new regions^12^. IRM is grounded in the principles of population genetics and benefits greatly from a basic understanding of the genetic mechanism of resistance^13^. For example, resistance allele frequency and the dominance of resistance can strongly shape the trajectory of the rise and spread of resistance^12^. Also, if the causal resistance gene(s) are known, genetic markers can be deployed to track resistance allele frequency across different regions and across time. This knowledge can lead to adaptive IRM strategies and better outcomes^14^. However, knowing these key facts requires a precise understanding of the gene(s) involved in resistance.

Fortunately, with respect to Cry1 family proteins, several studies have provided insights into their mode of action among lepidopteran insects. Early studies in *Heliothis virescens* demonstrated that Cry1Ac resistance was linked to mutations in a cadherin protein^15^. This receptor protein is embedded in the insect gut membrane and its wild-type form appears to interact with the Bt protein enabling toxicity^16^. Knockout mutations of this cadherin gene reduce or completely remove this interaction between Cry1Ac and its cadherin receptor, thereby conferring resistance^17–19^. More recently, mutations in ATP-binding cassette transporter proteins (ABC transporters) have also been shown to confer resistance to the Cry1 family proteins in *Bombyx mori*^20^, *Helicoverpa armigera*^21^, *Heliothis virescens*^22,23^, *Ostrinia nubilalis*^24^, *Spodoptera exigua*^25^, *Plutella xylostella*^26^, and *Trichoplusia ni*^26^. In each case resistance was linked to *ABCC2*, a member of the ABC transporter C family. In much the same way as with the cadherins, it is thought that Cry1 family proteins interact with *ABCC2* to initiate pore formation in the insect gut membrane^27^. When different truncation mutations render the ABCC2 protein non-functional, the loss of this interaction leads to resistance^23^.

The goal of the current study was to find the gene responsible for Cry1Fa and Cry1A.105 resistance in *S. frugiperda*, which in turn can enhance IRM by providing genetic markers for monitoring, with the ultimate goal of combatting Bt resistance. Because of the phylogenetically widespread involvement of *ABCC2* in resistance to the Cry1 family of proteins, we hypothesized that the reported resistance in *S. frugiperda* was likely due to a mutation in *ABCC2*. We tested this hypothesis on a Cry1Fa resistant colony collected from Puerto Rico, which also exhibits significant cross-resistance to Cry1A.105. We cloned the *ABCC2* gene and discovered that the resistant colony harbored two mutant alleles of *ABCC2*, each resulting in a predicted truncation of the protein. We show that a) both mutant alleles co-segregated with resistance in a simple Mendelian fashion in a F_2_ mapping population, b) one allele conferred resistance *in vitro* in a cell-based assay for toxin activity, and c) ABCC2 peptides from resistant *S. frugiperda* could not be detected in the insect gut membrane in a quantitative proteomics assay. Finally, we screened several field populations from the Puerto Rico and Brazil for the presence of one *ABCC2* mutant allele and detected its presence only in the populations from Puerto Rico.

Concurrent with this study, Banerjee *et al*.^28^, made many of the same discoveries, including the identification of one of the two resistance alleles we discovered. Our study also uncovered a unique resistance allele, making a total of two described alleles. Our work thus provides an immediate validation of the Banerjee *et al*.^28^ study, including arriving at the same conclusions using different methods. Our work is also complementary to Banerjee *et al*.^28^, because our geographic sampling differs from theirs and adds to our growing understanding of the nature of Cry1Fa and Cry1A.105 resistance in *S. frugiperda*.

## Results

### Establishment and characterization of a resistant colony

To understand the genetic mechanism of Cry1Fa and Cry1A.105 resistance in *S. frugiperda*, we first established a resistant colony. This colony was started with ~500 insects captured in non-Bt maize in Juana Diaz, Puerto Rico in 2010. Past reports indicated that *S. frugiperda* populations in Puerto Rico harbor high frequencies of resistance allele(s) to Cry1Fa and related Bt proteins^5^. The resistant colony (JuanaDiazR) was selected with the Cry1Fa toxin core (see Methods). After >50 generations of selection, characterization of the JuanaDiazR population suggested that it had >500-fold resistance to the Cry1Fa toxin core and 87-fold resistance to Cry1A.105, when compared to a susceptible colony (Supplementary Table S1).

### Identification of candidate resistance mutations in *S. frugiperda ABCC2*

**Figure 1:**
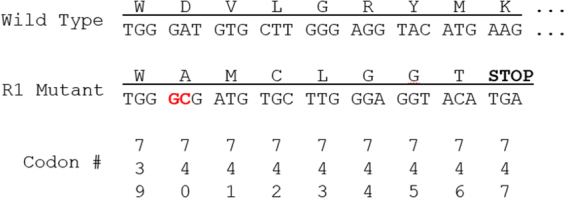
The diagram shows the amino acid (top) and codon (bottom) identities for the Wild Type and R_1_ mutant alleles of *SfABCC2*. The R_1_ mutant has a 2 bp GC insertion (highlighted in red) in the 740^th^ codon, which causes a premature stop codon to arise at codon 747.

**Figure 2:**
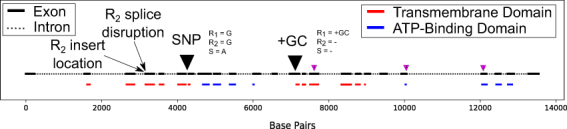
Schematic of representation of the *SfABCC2* gene. The two-marker system (SNP and +GC) used to diagnose the three segregating haplotypes is presented on the structure of the *SfABCC2* gene, along with the rubric used to identify each haplotype. This schematic applies to Family 1, however in Family 26 the S allele behaved dominantly, rather than codominantly, in R_2_S backgrounds, making it impossible to separate SS from R_2_S genotypes. The intron/exon structure of *SfABCC2* is given, and the protein domains are color-coded and shown below their corresponding exons. The location of the R_2_ insert and splice disruption sites on exon 4 is also marked. Finally, three numbered magenta triangles mark the location of the putatively paralogous peptides detected in in the resistant individuals using LC-MS/MS. Their numbering is as follows 1) FFDTNPSGR, 2) SSLISALFR, and 3) SKISIIPQEPVLFSASLR.

Test crosses between the JuanaDiazR resistant individuals and susceptible individuals obtained from Benzon Research (BenzonS) showed that the segregation of resistance among F_1_s was recessive and not sex linked (Supplementary Table S2). Moreover, after randomly mating F_1_s, their F_2_ progeny showed 3:1 susceptible:resistant phenotypic ratios, consistent with a recessive monogenic trait. Previous studies have shown that the *ABCC2* gene is a strong candidate as the binding partner for members of the Cry1 family of toxins^20,22–26^. With this knowledge, we took a candidate gene approach and cloned and sequenced the *S. frugiperda ABCC2* cDNA (*SfABCC2*) from the JuanaDiazR colony and compared it to a wild type allele from BenzonS. The JuanaDiazR population was found to harbor an allele with a two base pair + GC insertion 2,218 nucleotides from the start codon (Fig. 1). This mutation (referred to hereafter as R_1_) causes a frameshift which results in a premature stop codon 7 codons downstream from the insertion. The resulting protein is truncated to 747 amino acids, approximately half the length of the wild type *SfABCC2* allele. This truncation removes the final 6 (of 12) transmembrane domains of the protein (Fig. 2), along with the second ATP binding cassette, and is identical to the *SfABCC2mut* allele described in Banerjee *et al*.^28^.

We hypothesized that this large truncation of the *SfABCC2* gene in the R_1_ allele would render the translated protein a non-functional Cry1Fa and Cry1A.105 toxin receptor. To test this hypothesis, we expressed both the truncated R_1_ allele and the wild type (WT) allele in *S. frugiperda* Sf9 cells and evaluated whether either conferred susceptibility to the Cry1Fa toxin core or Cry1A.105 using the SYTOX Green fluorescence assay (Table 1). This assay measures cell death, with greater fluorescence indicating greater cell death. In both assays, the WT allele conferred far greater susceptibility than the R_1_ allele (Welch’s t-test p-value 0.006 and 0.003, respectively, both with 4 df). This result provided preliminary evidence that the R_1_ allele may obstruct the Bt toxin mode of action, making it a candidate allele for Cry1Fa and Cry1A.105 toxin resistance.

**Table 1:** SYTOX green fluorescence averages and standard deviations given in relative fluorescence units (RFU), for the wild type (WT) and R_1_ allele, and a GUS control.

Toxin Core	Target Receptor	Average RFU	St. Dev. RFU	N
Cry1A.105	WT	14928	1352	3
Cry1A.105	R_1_	1225	98	3
Cry1A.105	GUS control	493	108	3
Cry1Fa	WT	4283	550	3
Cry1Fa	R_1_	260	44	3
Cry1Fa	GUS control	88	8	3

Increased SYTOX Green fluorescence is associated with greater cell death.

In the SYTOX Green fluorescence assay we also detect a difference between the R_1_ allele and the GUS control (Welch’s t-test p-values < 0.003 for Cry1Fa and Cry1A.105, both with 4 df), suggesting that he R_1_ allele confers a degree of susceptibility in this assay, though it is an order of magnitude less than the WT allele. Finally, we note that the resistance observed in the SYTOX Green fluorescence assay was only 16.5- and 12.1-fold, respectively, for Cry1Fa and Cry1A.105. This is lower than the >500- and 87-fold resistance observed in the larval bioassay for these toxins. However, we do not expect of proportional response between these assays, as they measure different phenotypes (cell death vs. organismal death) and likely have different sensitivities.

We also investigated if the SfABCC2 protein was expressed in the larval brush border membrane-bound protein fraction from both the JuanaDiazR and BenzonS colonies. Brush border membrane proteins were subjected to tryptic digestion and LC-MS/MS analysis to identify candidate peptides matching SfABCC2. Table 2 lists all peptides identified in susceptible and resistant samples. Our hypothesis was that the truncated R_1_ allele from the JuanaDiazR strain would be unable to insert in the brush border membrane. This was true for 28 of the 31 peptides we detected (Table 2). Unexpectedly, three peptides were identified in JuanaDiazR (FFDTNPSGR, SSLISALFR, and SKISIIPQEPVLFSASLR). However, all three peptides occur after the R_1_ mutation in the JuanaDiazR strain (Table 2 and Fig. 2), and should not be expressed. This suggests they may come from another gene. In support of this hypothesis, we found two closely related genes (GSSPFP00023863001_OGS1.0 and GSSPFP00033000001_OGS1.0) residing on different genomic contigs of the corn variant *S. frugiperda* genome^29^. The latter of these two genes is a near perfect match for our translated clone of the wild type *SfABCC2* gene. Thus, the genome sequence provides evidence that the unexpected appearance of these three peptides in JuanaDiazR can be explained by expression from a closely related paralog in the ABCC family. A representative spectrum which shows the signal for the peptides that can be detected in the susceptible colony but not in the resistant colony is shown in Supp. Figure S1. The sequence map highlights the peptides which can be detected in the *Sf*ABCC2 protein. These results support the hypothesis that the truncated R_1_ allele is either absent from the brush border membrane or present at a level below LC-MS/MS detection.

**Table 2:** Proportion among eight individuals from the BenzonS and JuanaDiazR population that were positive for 31 peptides from the SfABCC2 protein.

Annotated Sequence	Susceptible Proportion Pos.	Resistant Proportion Pos.
[R].MSQVSVGDVAGGK.[L]	0.75	0
[K].YSPDDPPVLK.[D]	0.25	0
[K].VSEGGTNFSMGQR.[Q]	0.875	0
[R].ALEQVELKESIPALDYK.[V]	0.75	0
[R].ALEQVELK.[E]	0.625	0
[K].MYAWEKPFQLVVK.[A]	1	0
[K].DMGAMDELLPR.[S]	0.375	0
[R].SKISIIPQEPVLFSASLR.[Y]	1	0.125
[R].ENILFGLEYNVAK.[Y]	0.75	0
[R].QSGSLKWDVLGR.[Y]	0.25	0
[R].AYEMSALR.[K]	0.625	0
[K].SSLISALFR.[L]	0.75	0.875
[R].YWFEEVAIAEREDRDPSLWK.[A]	1	0
[R].IKLMSEIINGIQVIK.[M]	0.5	0
[R].IQGFLLLDER.[S]	0.75	0
[K].TSLLQLLLR.[E]	0.75	0
[R].SDIQITPK.[V]	0.375	0
[K].LMSEIINGIQVIK.[M]	0.625	0
[R].LSDITGSIKIDGLDTQGIAK.[K]	0.25	0
[R].FFDTNPSGR.[V]	0.875	0.5
[R].DVEEDDLIVPSK.[K]	0.75	0
[K].IAASSLLFR.[K]	0.625	0
[K].ILIMDEATANVDPQTDALIQK.[T]	0.125	0
[K].DLNFAIK.[S]	0.5	0
[K].IDGLDTQGIAK.[K]	0.5	0
[R].GVSLSGGQR.[AX]	0.625	0
[R].ASENLHNTIYEK.[L]	0.75	0
[K].VNATWADLNDNKEMTLK.[N]	0.25	0
[R].ILFEVAK.[T]	1	0
[K].SDDEEGEEKVQVLEAEER.[Q]	0.75	0
[K].LVNLLSNDVAR.[F]	0.25	0

### R_1_ mutation is not fixed in JuanaDiazR population

To determine the frequency of the R_1_ allele in the JuanaDiazR population, we developed a genetic marker assay to distinguish it from wild type and genotyped 48 randomly selected individuals from the JuanaDiazR colony. We found 2 WT/WT, 18 R_1_/WT, and 28 R_1_/R_1_ individuals. This results in an estimated R_1_ allele frequency of 77%, indicating that the R_1_ allele was not fixed in the JuanaDiazR population. If we assume R_1_ was the only resistance allele in the JuanaDiazR population and that it was recessive, then given the R_1_ allele frequency we would predict that approximately 41% of the individuals in the JuanaDiazR colony would be susceptible (i.e. either WT/WT or R_1_/WT genotypes) under Hardy-Weinberg equilibrium. This prediction was inconsistent with the results of our test crosses, and with the many generations of selection the JuanaDiazR colony had been through. We hypothesized that the JuanaDiazR population harbored two (or more) distinct resistance alleles, including the R_1_ allele we first identified and a second resistance allele (R_2_), which had not yet been functionally characterized. Moreover, if the hypothesized R_2_ allele is observed at approximately 23% allele frequency in the JuanaDiazR population, then the R_1_ and R_2_ alleles would be in Hardy-Weinberg Equilibrium (χ^2^ = 0.18, df = 2, p-value = 0.91), which would further support the existence of R_2_.

### Co-segregation of R_1_ and R_2_ alleles with Bt Protein resistance

**Figure 3:**
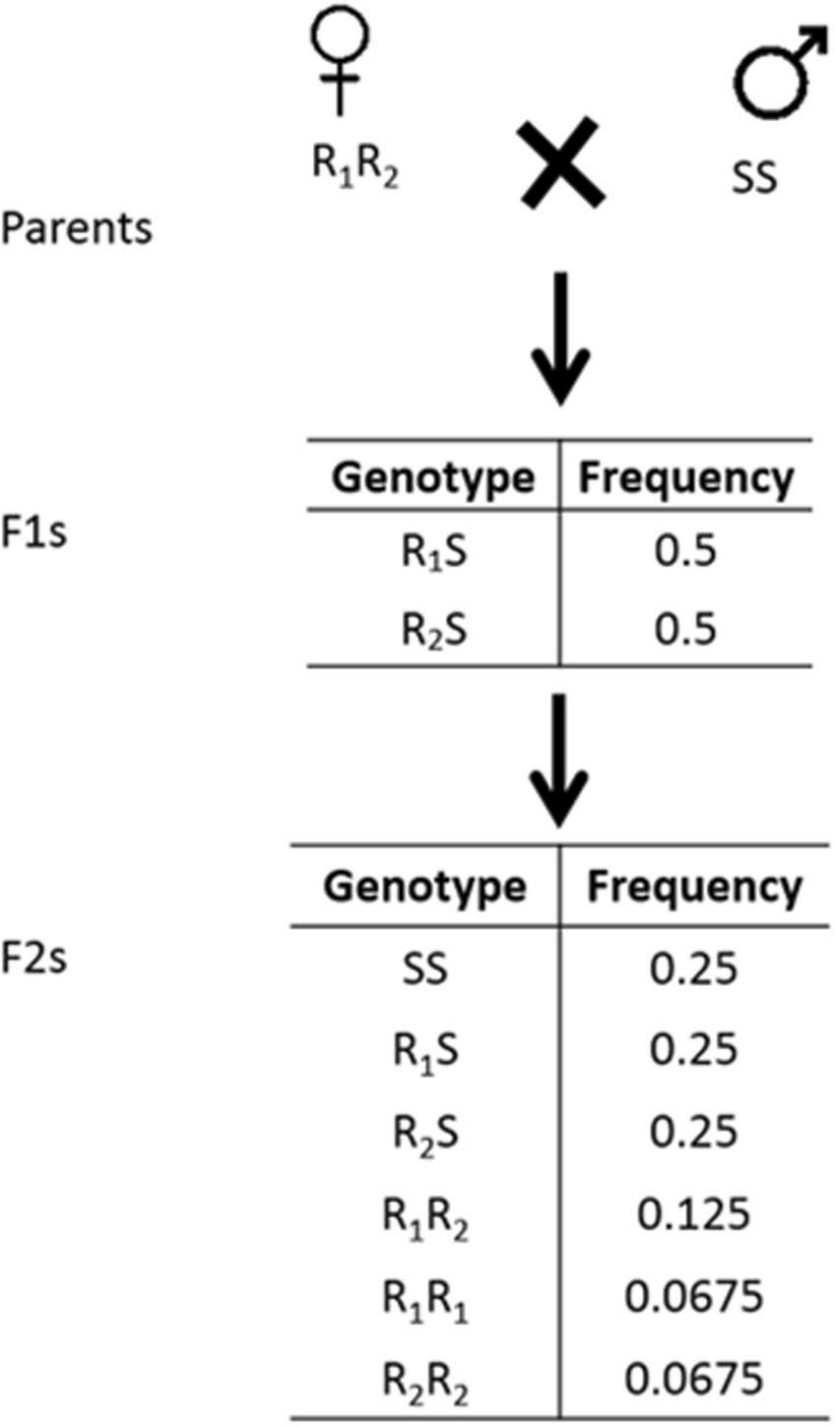
Mating scheme and expected genotype frequencies for Family 1 and 26, which had a heterozygous R_1_R_2_ resistant parent.

To test if the R_1_ allele and the hypothesized R_2_ allele were genetically linked with resistance, we constructed segregating populations through single pair matings by crossing a single JuanaDiazR resistant parent with a susceptible BenzonS parent (Fig. 3). In total 30 crosses were initiated and 16 successfully mated. After mating, the resistant colony parents from the 16 successful crosses were genotyped. For 14 pairs the resistant parent was the R_1_R_1_ genotype, and these pairs were discarded. We identified two pairs (#1 and #26) where the resistant parent was heterozygous for the R_1_ allele. These two resistant parents were putatively the R_1_R_2_ genotype. Within both families, F_1_ siblings were randomly bulk-mated to produce a segregating F_2_ population. The expected genotypic ratios of the F_2_ population can be found in Fig. 3. The key feature of this mating design is that it will produce R_1_R_1_, R_1_R_2_, and R_2_R_2_ genotypes, which allows us to determine if either allele co-segregates with resistance, and whether the alleles complement one another.

We lacked a genetic marker for the R_2_ allele, so we sampled both the susceptible and resistant parents and found a diagnostic SNP in *SfABCC2* gene. We do not believe that this SNP is itself associated with resistance, however, it allowed us to differentiate the R_2_ allele from the susceptible wild type allele. Also, because this SNP was only approx. 2,700 base pairs from the GC insertion site (Fig. 2), we can reasonably assume that it is unlikely for a recombination event to occur between these markers within the F_2_ mapping populations. This assumption allowed us to use these two codominant markers to diagnose the three allelic haplotypes (R_1_, R_2_, and S) segregating in the F_2_ mapping population (Fig. 2). The R_1_ haplotype was inferred present when an individual tested positive for the GC insertion at the R_1_ marker and had the G allele at the R_2_ marker. The R_2_ allele haplotype was inferred as the combination of the wild type allele at the R_1_ marker and the G allele for the R_2_ marker. Finally, the wild type susceptible (S) haplotype was inferred when the wild type allele was detected at R_1_ and the A allele at the R_2_ marker (Fig. 2). A further complication arose in family 26, where the marker assay for the S genotype was functionally dominant to the R_2_ genotype. For this family, we found an absence of the R_2_/S genotype. At the same time, we found a complementary excess of the S/S genotype (Table 3). A simple explanation for this result is that the S marker behaves dominantly in the R_2_/S background in family 26, confounding these genotypes. However, because this confounded class is highly susceptible (Table 3), there is no need to differentiate them into genotypic classes to interpret the experiment.

**Table 3:** F_2_ genotype and phenotype results for family #1 (top panel) and #26 (bottom panel).

Observed Genotype	Family 1			
	Resistant Individuals	Susceptible Individuals	Expected Freq	Observed Freq
R_1_R_1_	40	0	0.0625	0.0917
R_1_R_2_	57	0	0.125	0.1307
R_2_R_2_	24	0	0.0625	0.0550
R_1_S	5	125	0.25	0.2982
R_2_S	1	89	0.25	0.2064
SS	0	95	0.25	0.2179
Total	127	309		
**Observed Genotype**	**Family 26**			
	**Resistant Individuals**	**Susceptible Individuals**	**Expected Freq**	**Observed Freq**
R_1_R_1_	34	0	0.0625	0.0658
R_1_R_2_	61	2	0.125	0.1219
R_2_R_2_	40	1	0.0625	0.0793
R_1_S	1	107	0.25	0.2089
R_2_S	0	0	0.25	0
SS	2	269	0.25	0.5242
Total	138	379		

The genotype categories are listed on the rows, while phenotypic results are given in the second and third columns. The fourth and fifth columns list the Hardy-Weinberg expected genotype frequencies and the observed frequencies, respectively, for each genotype.

For families 1 and 26 we phenotyped 436 and 517 F_2_s, respectively, for their sensitivity to the Cry1Fa toxin core. As anticipated for a recessive trait, both F_2_ families segregated at approximately 3:1 susceptible:resistant ratios (Table 3). All individuals were genotyped and the R_1_, R_2_, and S haplotypes were inferred. In both families, the co-segregation of either allele with resistance was highly significant (Chi-Squared p-value < 0.001). Moreover, the R_1_R_2_ heterozygote class was resistant in both families, indicating that the R_1_ and R_2_ are complementary recessive resistance alleles of the *SfABCC2* gene.

In both families we see a small number of apparently misclassified individuals (e.g. R_1_S individuals who were phenotypically resistant). The most likely explanation for these individuals is that they are genotyping or phenotyping errors, similar to those seen by Coates and Siegfried^24^. However, in the diamondback moth (*Plutella xylostella*), Cry1Ac resistance was mapped to a locus containing several tightly linked candidate genes including five ABCC genes, and three MAP kinases, and two P450 genes^30^. Functional analyses were required to rule out the ABCC genes, and to implicate one of the MAP kinases. Analogous to this result, it is possible that resistance in *S. frugiperda* is conferred by a gene tightly linked to *SfABCC2*. Under this scenario, if all the misclassified individuals are taken at face-value, then we can infer that the true resistance gene must be approximately 0.63 cM from *SfABCC2*. There is strong evidence that *SfABCC2* is itself the likely resistance gene for Cry1Fa and Cry1A.105, however further work would be needed to rule out very closely linked genes^24^.

After establishing that R_2_ is a candidate resistance allele, we next investigated the mutational mechanism behind this allele. To do this we first isolated 10 F_2_ individuals with the R_2_/R_2_ genotype, along with 10 control individuals with the S/S and R_1_/R_1_ genotypes from family #1 and family #26. We then extracted RNAs, converted to cDNA, and cloned and sequenced the *SfABCC2* transcripts. cDNAs from all R_2_/R_2_ individuals exhibited a unique form of aberrant splicing approximately 600 nucleotides into the transcript (Figs 2 and S2). Most clones from the R_2_/R_2_ individuals were spliced slightly differently, but all cDNAs clones had a deletion of coding sequence relative to the wild type S/S individuals that created a missense mutation downstream (Supp. Figure S2). We next extracted genomic DNA from these same R_2_/R_2_ individuals, along with the S/S and R_1_/R_1_ controls, and identified an insertion near the start of the fourth exon unique to R_2_ (Figs 2 and S3). We were only able to gene walk 60 bp into this insertion before we hit a highly repetitive sequence and could no longer design unique PCR primers. Thus, we do not know the full size of the unique R_2_ insertion, but all S/S and R_1_/R_1_ control individuals lack it and we think it is likely that it is responsible for the aberrant splicing of R_2_. Similar to this scenario, aberrant splicing in a cadherin gene has been linked to Cry1Ac resistance in pink bollworm (*Pectinophora gossypiella*) in India^31^.

### Frequency of the R_1_ mutation in natural populations

Using the R_1_ marker described above, we genotyped several Puerto Rican and Brazilian *S. frugiperda* populations. In total, we screened 461 individuals (922 alleles) for presence of the +GC mutation that characterizes the R_1_ allele using the Taqman marker described above (Table 4). The R_1_ allele was only found in Puerto Rico, where it was at an intermediate to high frequency depending on location. The R_1_ allele was completely absent in the Brazilian populations tested, despite sampling 144 individuals, many coming from populations with appreciable phenotypic resistance to Cry1A.105 (Table 4). The absence of the R_1_ allele in Brazilian populations could indicate that either these populations have distinct, and yet undiscovered, mutations in *SfABCC2*, or possibly that resistance is not mediated by the mutations in the *SfABCC2* there. Further studies will be needed to determine which of these possibilities is correct.

**Table 4:** Geographic sampling of +GC allele. All Brazilian populations of *S. frugiperda* were subjected to Cry1A.105 assays and their survivorship is given. The column labled *N* gives the number of individuals genotyped.

Country	Location (State)	Year	*N*	+GC Allele Freq.	% Survivorship (Cry1A.105)
Brazil	Campo Grande (MS)	2016	10	0	57.5
Brazil	Campo Verde (MT)	2016	10	0	43.6
Brazil	Casa Branca (SP)	2016	10	0	7.8
Brazil	Casa Branca (SP)	2016	10	0	18.9
Brazil	Ivatuba (PR)	2016	10	0	15.0
Brazil	Londrina (PR)	2016	10	0	52.6
Brazil	Não-me-Toque (RS)	2016	10	0	4.0
Brazil	Palotina (PR)	2016	10	0	25.3
Brazil	Ponta Grossa (PR)	2016	10	0	31.2
Brazil	Rio Verde (GO)	2016	10	0	25.9
Brazil	Santa Helena de Goiás (GO)	2016	10	0	19.0
Brazil	Santo Ângelo (RS)	2016	10	0	56.3
Brazil	Sapezal (MT)	2016	4	0	46.0
Brazil	Seara (SC)	2016	10	0	0.0
Brazil	Água Fria de Goiás (GO)	2016	10	0	38.8
USA	Benzon-JuanaDiazR (PR)	2010	48	0.77	NA
USA	Juana Diaz (PR)	2015	177	0.88	NA
USA	Isabella (PR)	2015	49	0.48	NA
USA	Unknown (PR)*	2011	43	0.32	NA

^*^Samples provided by Fengneng Huang^4^.

## Discussion

Bt crops represent one of the most rapidly adopted technologies for managing insect pests in maize, cotton, and soybean. The evolution of insect resistance to these crops is a major threat to their sustainability. Indeed, in two decades of Bt crop of cultivation, several insect species have developed resistance resulting in field control failures^32,33^. *S. frugiperda* is one such insect. It has developed resistance to Bt maize expressing the Cry1Fa protein in Puerto Rico^5^, the US mainland^4^, and Brazil^11^, with cross-resistance to Cry1A.105. Many studies have been conducted on various aspects of resistance to Cry1 family proteins in *S. frugiperda*, including inheritance, fitness costs, frequency of resistant alleles, resistance mechanism, allelic variation, and cross resistance to other Bt proteins^4–6,8,9,11,34–36^. Recently, Banerjee *et al*.^28^ performed competitive binding assays demonstrating that *SfABCC2* is a receptor for Cry1F1. Here, we report confirmation of *SfABCC2* as a receptor protein for Cry1Fa and Cry1A.105, and identify mutations causing resistance in a population collected from Juana Diaz, Puerto Rico.

Receptors play a significant role in mode of action of Bt proteins. Several classes of proteins (cadherins, ABC transporters, aminopeptidases, alkaline phosphatase) have been reported as Bt protein receptors in insects^23^. Mutations in these receptor proteins leading to reduced or no Bt protein binding have been reported to cause resistance in many insects^22–25,28^. Previous studies by Jakka *et al*.^34^ with Cry1Fa resistant *S*. *frugiperda* reported reduced binding of Cry1Fa, Cry1Ab and Cry1Ac but not Cry1Ca compared to a susceptible colony. They also observed an associated reduction in a membrane-bound alkaline phosphatase (ALP), which suggested that this gene might be a candidate for the Cry1Fa and Cry1A.105 resistance locus.

In addition to ALP, the *ABCC2* gene was a strong candidate due to its involvement in Cry1 protein family resistance in diverse lepidopteran species^20–24,26^, including *S. exigua*^24,25^, a close relative of *S. frugiperda*. Because of this strong prior evidence, we decided to focus first on *SfABCC2*, and as demonstrated above, we found compelling evidence that when mutated it is responsible for Cry1Fa and Cry1A.105 resistance.

Concurrent to our studies, researchers from J. L. Jurat-Fuentes’ laboratory have reported the identification of *SfABCC2* as a receptor protein for Cry1Fa and mutations resulting in field resistance^28^. These authors also discovered a mutant allele called *SfABCC2mut*, which is the same as our R_1_ allele, (i.e. a two base pair GC insertion at nucleotide 2,218). Consistent with our findings, these authors established that this mutation disrupts translation, creating a partial protein which does not function as a Cry1Fa or Cry1A.105 receptor. The results from our work and those of Banerjee *et al*.^28^ have come to the same conclusion, despite working with populations collected at different times, selected/maintained differently and studied with different methods.

Furthermore, where Banerjee *et al*.^28^ explore the temporal and spatial distribution of resistance alleles in North America, our study explores their spatial distribution in both North and South America, adding additional geographical data. Similar to the conclusions from Banerjee *et al*.^28^ in North America, our results show an absence of the *SfABCC2mut*/R_1_ resistance allele in samples from Brazil. Together these results suggest that Cry1Fa and Cry1A.105 resistance in *S. frugiperda* may develop repeatedly in local populations, rather than infrequently and spread to new areas via long distance migration^4^. The resistance alleles found in Puerto Rico may be private alleles that came to high frequency in this island population. This is consistent with results from other species. For example, there are three known mutations in the *ABCa2* gene which cause resistance to Cry2Ab in *Helicoverpa armigera*^37^, and cadherin mutations observed in laboratory developed Cry1Ac-resistant *Pectinophora gossypiella* in Arizona^38^ were different from those observed in field resistant colonies in India^31^. Consistent with these past studies, our work, and that of Banerjee *et al*.^28^, demonstrates that strong resistance to Cry1Fa and Cry1A.105 can evolve through simple loss-of-function mutations to *SfABCC2*. There are likely a large number of mutations that can render *SfABCC2* nonfunctional as a Bt receptor, so it is not surprising to have new resistance alleles being created at a steady rate in nature. The occurrence of many resistant alleles, even in the same gene, limits the utility of DNA marker-based detection methods for resistance monitoring. Therefore, alternative methods, such as protein-based assays to identify disrupted receptor proteins or whole gene sequencing techniques to identify new mutations should be explored.

## Methods

### Insects

Eggs from a susceptible laboratory population were received from Benzon Research Laboratory (Carlisle, PA). A resistant colony was established in 2010 through larvae collected on non-Bt maize from Juana Diaz, Puerto Rico (JuanaDiazR). This colony was crossed with the susceptible colony (BenzonS) and selected using TIC842, a Cry1Fa-like Bt protein consisting of toxin domains I-III from Cry1Fa and a protoxin domain from Cry1Ac. Although this is a chimeric protein, insects are effectively selected against the activated Cry1Fa toxin core as the Cry1Ac protoxin domain is cleaved within in the insect gut. Similarly, the Bt event TC1507 expresses a truncated Cry1Fa, exposing insects to only activated Cry1Fa toxin core^39^, meaning that TIC842 presents the same Cry1Fa toxin core to the insect gut as does TC1507 and represents an equivalent selection pressure. For these reasons we refer to TIC842 as the Cry1Fa toxin core throughout this manuscript. The bouts of selection were conducted in alternative generations through exposing larvae to diet surface-treated with TIC842 at 2.0 µg/cm^2^ concentration for 7 D and rearing surviving second instar larvae to the pupal stage on non-treated diet. Characterization of this strain indicated >579-fold resistance to TIC842 (*i.e*. the Cry1Fa toxin core) and >87-fold cross-resistance to Cry1A.105 (Supplemental Table S1). Resistance was stable when insects were removed from selection for up to two generations (Supplemental Figure S4) and showed recessive inheritance (Supplementary Table S2).

### Cloning and Sequencing *ABCC2* from BenzonS and JuanaDiazR Colony

Gut tissues were dissected from 5 third instar *S. frugiperda* larvae from both populations (JuanaDiazR and BenzonS) and pooled within population. RNA was extracted from each pool following the AllPrep DNA/RNA mini kit manual from Qiagen (Hilden, Germany). First strand cDNA was synthesized using the GeneRacer^TM^ Kit from Invitrogen. *ABCC2* coding sequence was amplified from cDNA with primers given in Supp. Table S3. PCR products were cloned into the pIEx/Bac^TM^-3 vector from Novagen (Damstadt, Germany). Plasmid DNA was extracted by following QIAprep Spin Miniprep Kit manual (Qiagen, Hilden, Germany), and sequenced by Sanger sequencing. Sequences were translated into amino acids and manually inspected for mutations. These cDNA sequences can be found on Genbank under accessions MG387043-MG387070.

### Recombinant Baculovirus Generation and Cell-Based Assay

Recombinant baculovirus for mutant and wildtype *ABCC2* expression in Sf9 cells were generated using the BacMagic^TM^-3 DNA kit (Novagen, Damstadt, Germany). Sf9 cells were cultured in SF900III SFM medium in a refrigerated incubator/shaker at 27 °C and 150 rpm. The culture was diluted to a cell density of 5 × 10^5^ cells/ml in growth medium and were seeded on 96 well black cell culture plates (catalog# 165305, Thermo Fisher Scientific, Waltham, MA, USA) with 100 ul/well. The seeded plates were incubated in a 27 °C incubator for cell recovery. To express *ABCC2*, the growth medium was removed after cell recovery, and 100 ul recombinant baculovirus (1 µl virus plus 99 ul SF900III SFM) was added to each well. After 48 h expression in 27 °C incubator, the medium was changed to the toxin mixture (50 ppm toxin + 2 µM SYTOX Green (catalog # S7020, Thermo Fisher Scientific, Waltham, MA, USA)) in unsupplemented Grace’s medium (catalog # 11595030, Thermo Fisher Scientific, Waltham, MA, USA). Cells were incubated with toxins for 4 h in 27 °C incubator, and then SYTOX Green fluorescence intensity was read by a CLARIOstar microplate reader (BMG Labtech, Offenburg, Germany). Recombinant baculovirus expressing β-glucuronidase (GUS) was used as virus control.

### Taqman Marker Design

To genotype *ABCC2* alleles, we used Taqman genotyping assays (Thermo Fisher Scientific, Waltham, MA). Genotyping primer sets are given in Supp. Table S3. All genotype assays were carried out following the manufacturers instructions.

### Cloning R_2_ allele

We extracted RNA from 10 individuals from the F_2_ mapping population with the R_2_R_2_ genotype and converted to cDNA as described above. cDNAs were then cloned and sequenced using Sanger sequencing. These cDNA sequences can be found on Genbank under accessions MG387043-MG387070.

### Phenotypic assay for resistance

Lepidopteran larvae typically have alkaline gut pH which will change to acidic pH when midgut epithelial cells are damaged (*e.g*. Bt protein interaction with receptors) leading to exchange of cell contents with gut lumen. We developed a highly sensitive phenotyping assay using thymol blue, an indicator dye to measure midgut pH in *S. frugiperda*. The larvae are fed on the diet containing thymol blue and those that have intact midguts will maintain alkaline pH and retain the blue color in the midgut while larvae that have a compromised gut will lose the blue color and appear to have clear midguts. Using this assay one can identify resistant and susceptible larvae based on their response to given Bt protein.

Preliminary experiments were conducted to identify a diagnostic concentration to separate resistant and susceptible larvae. In this study, larvae were first exposed to diet containing thymol blue for 24 h and then transferred to diet containing thymol blue and surface treated with 11 concentrations of TIC842 ranging from 5.26 to 5.26 × 10^−20^ µg/cm^2^ for 4 h. Larvae were scored visually for the midgut coloration and the experiment was continued for 5 D, and scored for mortality to correlate early and endpoint responses. Based on these results (Supp. Figure 5), 0.526 µg/cm^2^ was selected as a diagnostic concentration. Following this protocol, F_2_ larvae from mapping populations were exposed to diet containing thymol blue for 24 h and then to diet containing thymol blue and surface treated with TIC842 at 0.526 µg/cm^2^ for 4 h. Phenotyped larvae were transferred to untreated diet, reared until third instar and used for genetic studies.

### Brush Border Membrane Preparation for Mass Spectrometry

Brush border membrane vesicles were isolated from neonates following Wolfersberger *et al*.^40^. Neonates were homogenized with a polytron mister in buffer containing 5 mM TRIS (pH 7.4), 50 mM sucrose with lipase inhibitor, PMSF and protease inhibitor cocktail at 16,000 rpm for three 10 sec pulses. In presence of CaCl_2_, the samples were transferred and centrifuged at 4300 g and 4 °C for 30 min. The supernatant was filtered through 4 layers of cheese cloth and centrifuged at 27,000 g and 4 °C for 30 min. The pellet was dried and resuspended in sucrose buffer. Protein concentration of the 6 BBM samples was determined by Bradford assay (Bio-Rad, Hercules, CA, USA) according to manufacturer’s protocol. The BBM were quality tested using alkaline phosphatase and leucine aminopeptidase enzyme assays. 50ug brush border membrane protein sample was reduced and digested with trypsin using proteome CEM Discover Proteomics System (Matthews, NC, USA). The resulting peptides were acidified with formic acid to pH < 3 to stop the reaction.

Data Acquisition by Nano-Liquid Chromatography Mass Spectrometry and Protein Identification. Peptides were analyzed on an Ultimate 3000 nanoLC system connected with nanospray Q-Exactive HF Orbitrap MS (Thermo Fisher Scientific, Waltham, MA, USA) operated in Full MS/ddMS^2^ mode. The data were collected with the installed Xcalibur software (Thermo Fisher Scientific, Waltham, MA, USA). The binary mobile phase was used consisting of 0.1% formic acid in water or 80% acetonitrile. A flow rate of 5 µl/min was used to load the sample onto a C18 PepMap trap column (300 μm ID × 5 mm, Thermo Fisher Scientific, Waltham, MA, USA). The peptides were eluted from the trap column and separated at a flow rate of 300 nL/min on a C18 Tip column (75 μm ID × 150 mm, Acclaim PepMap@RSLC, Thermo Fisher Scientific, Waltham, MA, USA) with a spray voltage of 1.9 kV. The gradient was run with 10−30% B in 40 min for elution. Full-scan mass spectra were acquired in the Orbitrap over a mass range of 400−1,600 m/z with a resolution of 120,000 at AGC target 3 × 10^6^. A lock mass function was used to obtain high mass accuracy. The 12 most intense precursor ions were selected for collision-induced fragmentation with normalized collision energy of 27% with a resolution of 15,000 with AGC target as 1 × 10^5^. For each sample, the injection volume was adjusted per the protein assay to load 1 µg on column. Three technical replicates were applied with each sample.

Proteins were identified by the Proteome Discoverer (version 1.4; Thermo Fisher Scientific, Waltham, MA, USA). The *S. frugiperda* protein database (1,786 sequences) from uniport was combined with a *S. frugiperda* ABCC2 (SfABCC2) protein sequence (Supp. Figure S1) and a reversed decoy database was used for comparison. Data files were generated from acquired raw data files with Thermo Xcalibur. The protein identifications were filtered in Proteome Discoverer retaining only proteins that contained at least three peptides with XCorr scores above the threshold. The data include only rank 1 peptides and peptides in the top scored proteins. Parameters used in searches were as follows: Carbamidomethylation of cysteine and oxidation of methionine were set as modifications. Trypsin was specified as the proteolytic enzyme, and one missed cleavage was allowed. Peptide mass tolerance was set at 10 ppm, fragment mass tolerance was set at 0.6 Da, and peptide charge was set at + 2, +3, and +4. False discovery rates for peptide identification of all searches were less than 5.0%. For three biological samples each, three technical replicates were applied.

## Electronic supplementary material

Supplementary Information
