# Supplementary material for: Mutational disruption of the ABCC2 gene in fall armyworm, Spodoptera frugiperda, confers resistance to the Cry1Fa and Cry1A.105 insecticidal proteins
Source: Sci Rep. 2018 May 8;8:7255. doi: 10.1038/s41598-018-25491-9 (PMC5940765; doi:10.1038/s41598-018-25491-9)
Supplement: Supplementary file 1 — Supplementary Information [file 41598_2018_25491_MOESM1_ESM.pdf]

Mutational disruption of the *ABCC2* gene in fall armyworm, *Spodoptera frugiperda*, confers resistance to the Cry1Fa and Cry1A.105 insecticidal proteins[1]

Authors: Lex Flagel[2,6], Young Wha Lee[2,6], Humphrey Wanjugi[2], Shilpa Swarup[2], Alana Brown[2], Jinling Wang[3], Edward Kraft[3,7], John Greenplate[2], Jeni Simmons[4], Nancy Adams[4], Yanfei Wang[2], Samuel Martinelli[5], Jeffrey A. Haas[2], Anilkumar Gowda[2], and Graham Head[5,*].

[1]Dedicated to the memory of John Greenplate. This work would not have been possible without John's intelligence, experience, and friendship.

[2]Monsanto Company, Chesterfield, Missouri

[3]Monsanto Company, Cambridge, Massachusetts

[4]Monsanto Company, Union City, Tennessee

[5]Monsanto Company, Creve Coeur, Missouri

[6]These authors contributed equally

[7]Present address: Genentech,, South San Francisco, California

*Correspondence to graham.p.head@monsanto.com

Supp. Figure S1

A) LC/MS/MS analysis of representative peptides identified as belonging to SfABCC2 in the susceptible BenzonS (top panel) and JuanaDiazR (bottom panel). MS/MS sequencing data of peptides identified in the eluted fraction in 9 out of 9 runs of the BenzonS colony samples and 0 out of 9 runs in JuanaDiazR colony samples. B) The sequence map of the identified peptides are highlighted in green on the *Sf*ABCC2 peptide sequence.

A)

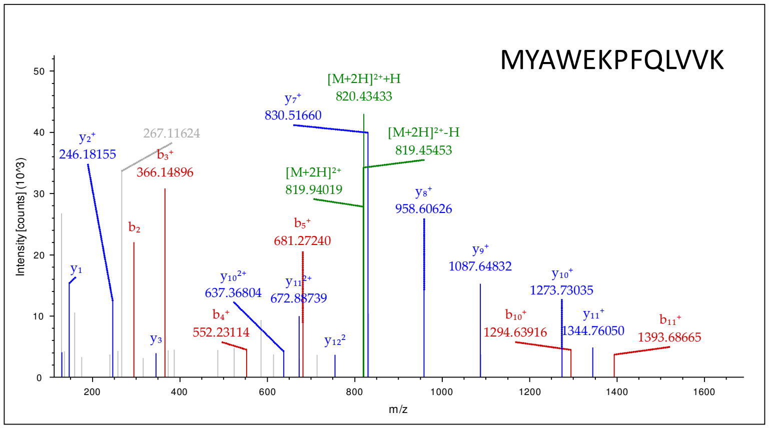

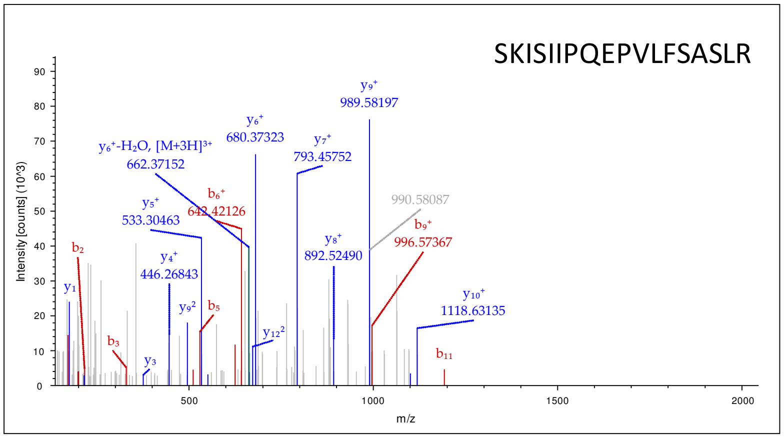

B)

MMDKSNKNTA SNGTAVGEPK ERVRKKPNIL SRIFVWWIFP VLITGNKRDV EEDDLIVPSK KFNSERQGEY FERYWFEEVA
IAEREDRDPS LWKAMRRAYW LQYMPGAIFV LLISGLRTAQ PLLFSQLLSY WSVDSEMSQQ DAGLYALAML GINFITMMCT
HHNNLFVMRF SMKVKIAASS LLFRKLLRMS QVSVGDVAGG KLVNLLSNDV ARFDYAFMFL HYLWVVPLQV GVVLYFVYDA
AGWAPYVGLF GVIILIMPLQ AGLTKLTGVV RRMTAKRTDK RIKLMSEIIN GIQVIKMYAW EKPFQLVVKA ARAYEMSALR
KSIFIRSMFL GFMLFTERSV MFLTVLTLAL TGNMISATLI YPIQQYFGII TMNVTLILPM AFASFSEMLI SLERIQGFLL
LDERSDIQIT PKVVNGAGSK LFNNSKKEGG LETGIVLPTK YSPTEANIAR PMQDEPNMAD YPVQLNKVNA TWADLNDNKE

MTLKNISLRV RKNKLCAVIG PVGSGKTSLL QLLLRELPVT SGNLSISGTV SYASQEPWLF PATVRENILF GLEYNVAKYK
EVCKVCSLLP DFKQFPYGDL SLVGERGVSL SGGQRARINL ARAVYREADI YLLDDPLSAV DANVGRQLFD GCIKGYLSGK
TCILVTHQIH YLKAADFIVV LNEGSVENMG SYDELMKTGT EFSMLLSDQA SEGSDTDKKE RPAMMRGISK MSVKSDDEEG
EEKVQVLEAE ERQSGSLKWD VLGRYMKSVN SWCMVVMAFL VLVITQGAAT TTDYWLSFWT NQVDGYIQTL PEGESPNPEL
NTQVGLLTTG QYLIVHGSVV LAIIILTQVR ILSFVVMTMR ASENLHNTIY EKLIVAVMRF FDTNPSGRVL NRFSKDMGAM
DELLPRSMLE TVQMYLSLAS VLVLNAIALP WTLIPTTVLM FIFVFLLKWY INAAQAVKRL EGTTKSPVFG MINSTISGLS
TIRSSNSQDR LLNSFDDAQN LHTSAFYTFL GGSTAFGLYL DTLCLIYLGI IMSIFILGDF GELIPVGSVG LAVSQSMVLT
MMLQMAAKFT ADFLGQMTAV ERVLEYTKLP TEENMETGPT TPPKGWPSAG EVTFSNVYLK YSPDDPPVLK DLNFAIKSGW
KVGVVGRTGA GKSSLISALF RLSDITGSIK IDGLDTQGIA KKLLRSKISI IPQEPVLFSA SLRYNLDPFD NYNDDDIWRA
LEQVELKESI PALDYKVSEG GTNFSMGQRQ LVCLARAILR SNKILIMDEA TANVDPQTDA LIQKTIRKQF ATCTVLTIAH
RLNTIMDSDR VLVMDQGVAA EFDHPYILLS NPNSKFSSMV KETGDNMSRI LFEVAKTKYE SDSKTA*

Supp. Figure S2:

Splicing pattern among R_2, R_1, and S genotypes. Below the first 18 cDNA sequences show abberate splice patterns among R_2/R_2 genotypes. The region of abberant splicing spans from approx 580 to 710 bp in the transcript (black box). Below that are cDNA clones from R_1/R_1 and S/S genotypes, which all have identical patterns, and maintain a proper reading frame (red box). The cDNA sequences below correspond to Genbank accessions MG387043-MG387070. The breakdown of clones is as follows:

Fam1 R1R1: 2
Fam1_R2R2: 14
Fam1 SS: 4
Fam26 R2R2: 1
Fam26 SS: 2
Fam26_R1R1: 1
Fam26 R2R2: 3

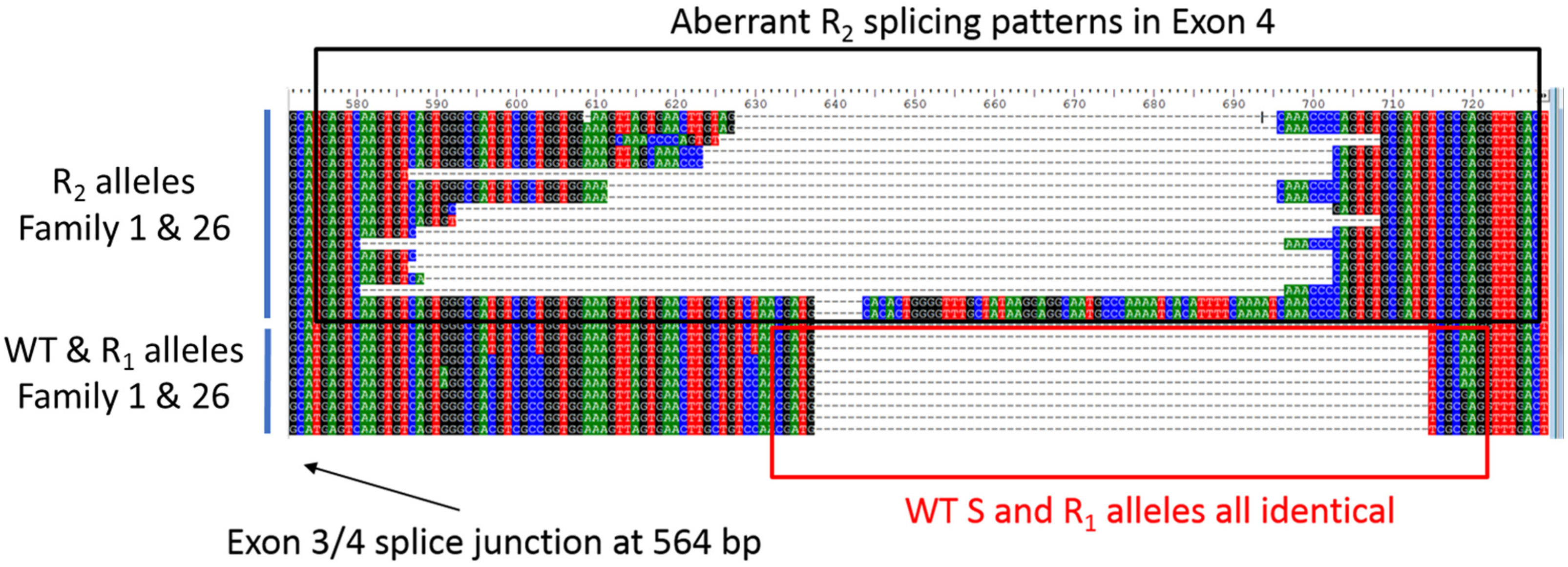

Supp Figure S3:

Three genomic DNA clones from a R_2/R_2 individual are aligned to a clone from a wild type individual. At position 631 in the aligment the two sequences change when going from 3` to 5`. This is evidence of an insertion in the genomic DNA from the R_2/R_2 individual that is absent from the wild type individual. The sequences below correspond to Genbank accessions MG387070-MG387074.

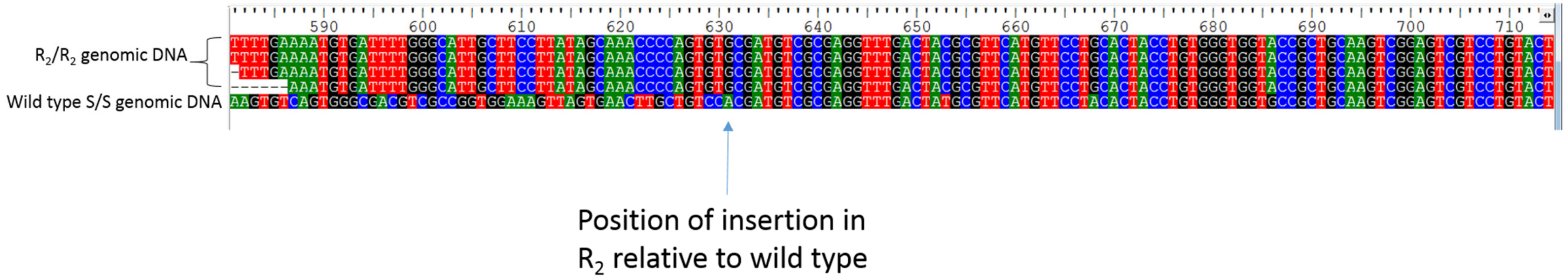

Supp. Figure S4: Stability of Cry1Fa toxin core resistance in *S. frugiperda* when removed from selection pressure for one (a) or two (b) generations.

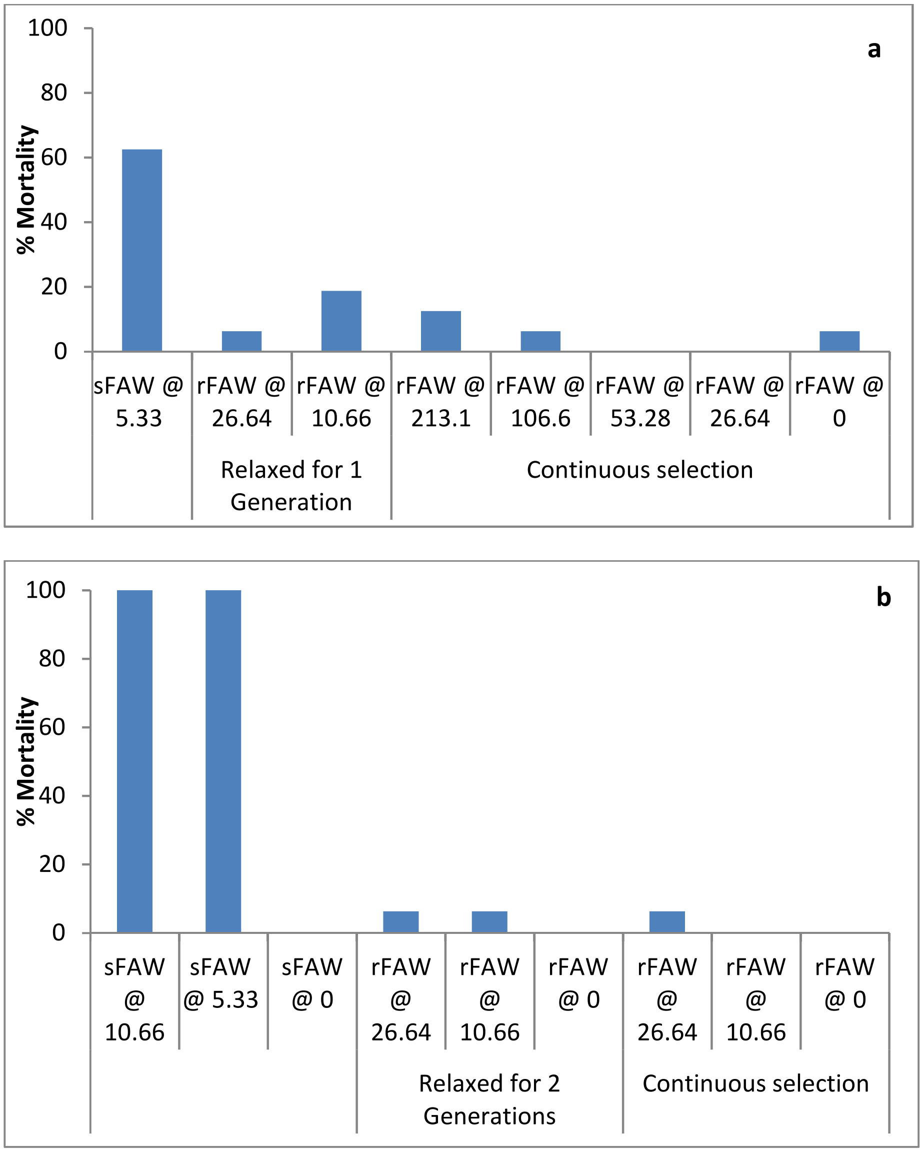

Supp. Figure S5: Response (a) and mortality (b) of Cry1Fa toxin core resistant and susceptible *S. frugiperda* populations in the Cry1Fa toxin core phenotyping assay.

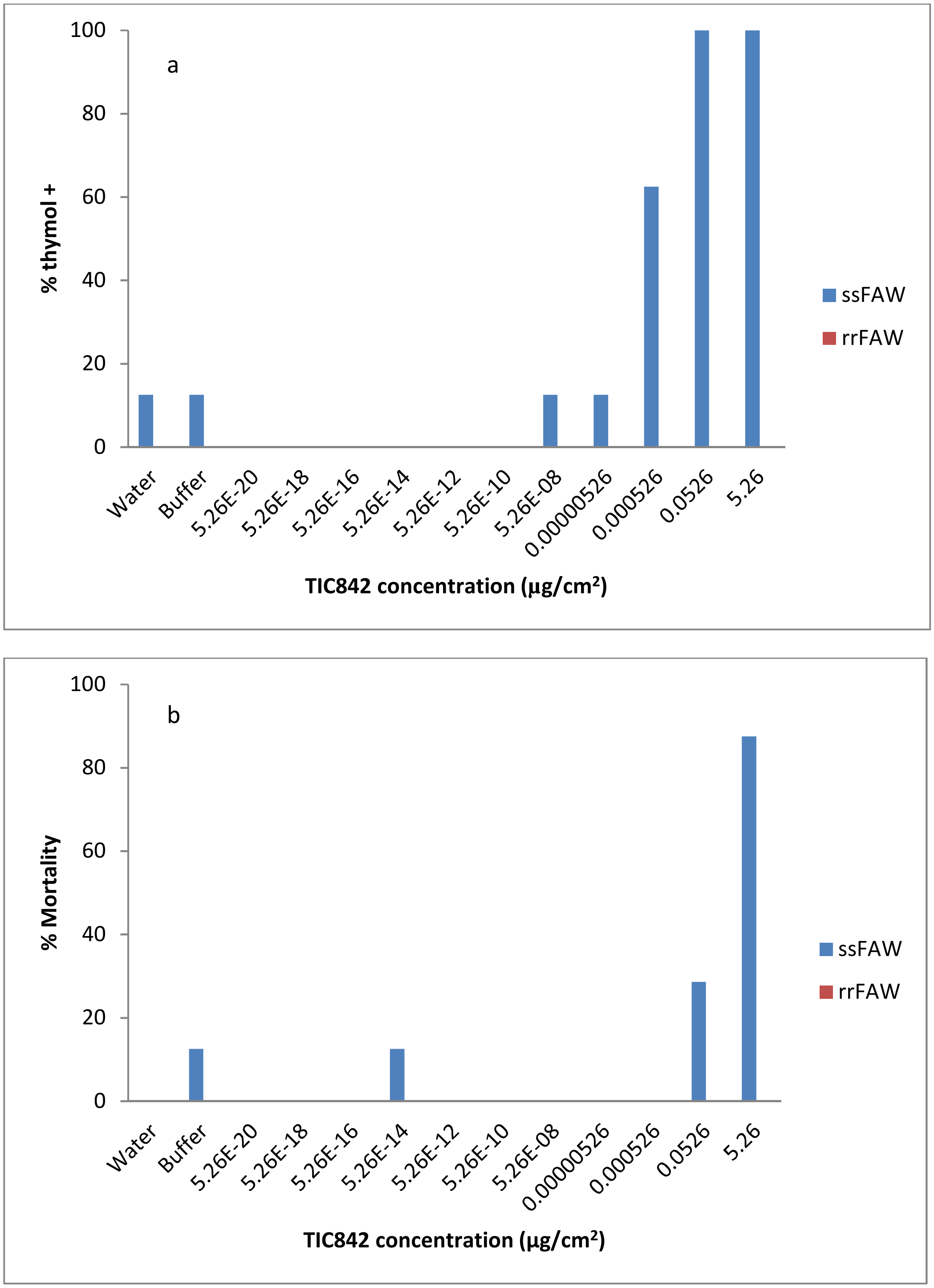

Supp. Table S1: Level of resistance and cross resistance to Cry1Fa toxin core and Cry1A.105 in the resistant and susceptible *S. frugiperda* populations, respectively.

Protein	Strain	LC_{50} (fiducial limits)	Slope ± SE	Resistance ratio
Cry1Fa toxin core	Resistant	>21,310*		>579-fold
	Susceptible	36.80 (28.53 - 49.53)	2.22 ± 0.28	1
Cry1A.105	Resistant	>21,310		>87-fold
	Susceptible	243.79 (205.64 - 301.61)	2.58 ± 0.27	1

* 9.38 and 25% mortality at 21,310ng/cm^2 for Cry1Fa toxin core and Cry1A.105, respectively

Supp. Table S2: Response of Cry1Fa/Cry1A.105 toxin resistant, susceptible and progenies of reciprocal crosses of these two populations to Cry1Fa toxin core.

Strain	LC_{50} (fiducial limits)	Slope	Chi Sq	Resistance ratio
Resistant	>21,310*			>740
rMale X sFemale	125.73 (82.05 - 193.93)	0.67 ± 0.35	<0.0001	4.4
sMale X rFemale	98.03 (57.01 - 158.23)	0.67 ± 0.36	<0.0001	3.41
Susceptible	28.77 (9.06 - 52.74)	1.12 ± 0.26	<0.0001	1

* 0% mortality at 21,310 ng/cm^2

Supp. Table S3: Primer sequences used in this study

Assay	Forward Primer Seq.	Reverse Primer Seq.	VIC Probe Sequence	FAM Probe Sequence	Resistant	Susceptible
SfABCC2 cDNA	ATGATGGACAAATCTAATAAAAATACCGCGTC	CTAAGCGGTTTTGGAATCACTTTCAT	NA	NA	NA	NA
R_1/WT Genotype	TGGAGGCCGAAGAGAGACA	AGGAGTTGACTGACTTCATGTACCT	CAAGCACATCGCCCACT	CCAAGCACATCCCACT	+GC	-
R_2/WT Genotype	AGGGTAACGTTCATTGTGATAATACC	CGCTGACTGGAAACATGATTAGTG	CCATCCAACAGTACTTC	CATCCAACAATACTTC	GG	AA
